# Editorial: The roles of ion-induced cell death in cancer treatment: volume II

**DOI:** 10.3389/fphar.2023.1289829

**Published:** 2023-09-15

**Authors:** Kui Zhang, Zhi-Yao He, Abhimanyu Thakur, Xin Hu, Isha Gaurav, Zhijun Yang, Zhijie Xu, Guangzhao Pan

**Affiliations:** ^1^ State Key Laboratory of Resource Insects, Medical Research Institute, Southwest University, Chongqing, China; ^2^ Department of Pharmacy, West China Hospital, Sichuan University, Chengdu, Sichuan, China; ^3^ The Pritzker School of Molecular Engineering, Ben May Department for Cancer Research, University of Chicago, Chicago, IL, United States; ^4^ School of Chinese Medicine, Hong Kong Baptist University, Hong Kong, Hong Kong SAR, China; ^5^ Changshu Research Institute, Hong Kong Baptist University, Changshu Economic and Technological Development (CETD) Zone, Changshu, China; ^6^ Department of Pathology, Xiangya Hospital, Central South University, Changsha, Hunan, China; ^7^ Zhejiang Cancer Hospital, Hangzhou Institute of Medicine (HIM), Chinese Academy of Sciences, Hangzhou, Zhejiang, China; ^8^ Key Laboratory of Prevention, Diagnosis and Therapy of Upper Gastrointestinal Cancer of Zhejiang Province, Hangzhou, Zhejiang, China

**Keywords:** ion homeostasis, cell death, ferroptosis, cuproptosis, PANoptosis, cancer

## Introduction

Ion homeostasis plays a vital role in cellular functions, primarily by maintaining stable ion concentrations. An imbalance in this equilibrium can result in irreversible cell damage by modulating various intracellular signaling pathways. Such disruptions have critical implications on tumor development and the effectiveness of therapeutic interventions. Essential ions, such as calcium, potassium, sodium, chloride, magnesium, hydrogen, bicarbonate, phosphate, iron, zinc, copper, manganese, iodide, fluoride, and sulfate, are collectively required for maintaining cellular function, physiological balance, and overall homeostasis in organisms. The dysregulation of essential ion concentrations can have several negative consequences for cells, including cell death, abnormal cell growth, and reduced function.

Ferroptosis (Lei et al., 2022) and curoptosis (Xie et al., 2023), the iron and copper-induced cell death pathways, are novel and emerging as promising targets for cancer research and therapy. However, the current state of therapeutic strategies, grounded in our understanding of ion-driven cell deaths, especially about the iron and copper, has not fully met the desired clinical efficacy. It underscores the urgent need for in-depth research into the molecular underpinnings and biological impacts of ion-induced cell death in the cancer therapy. Advancements in this domain promise to pioneer new, more effective therapeutic approaches against cancer.

## Ion-induced ferroptosis in cancer

**FIGURE 1:**
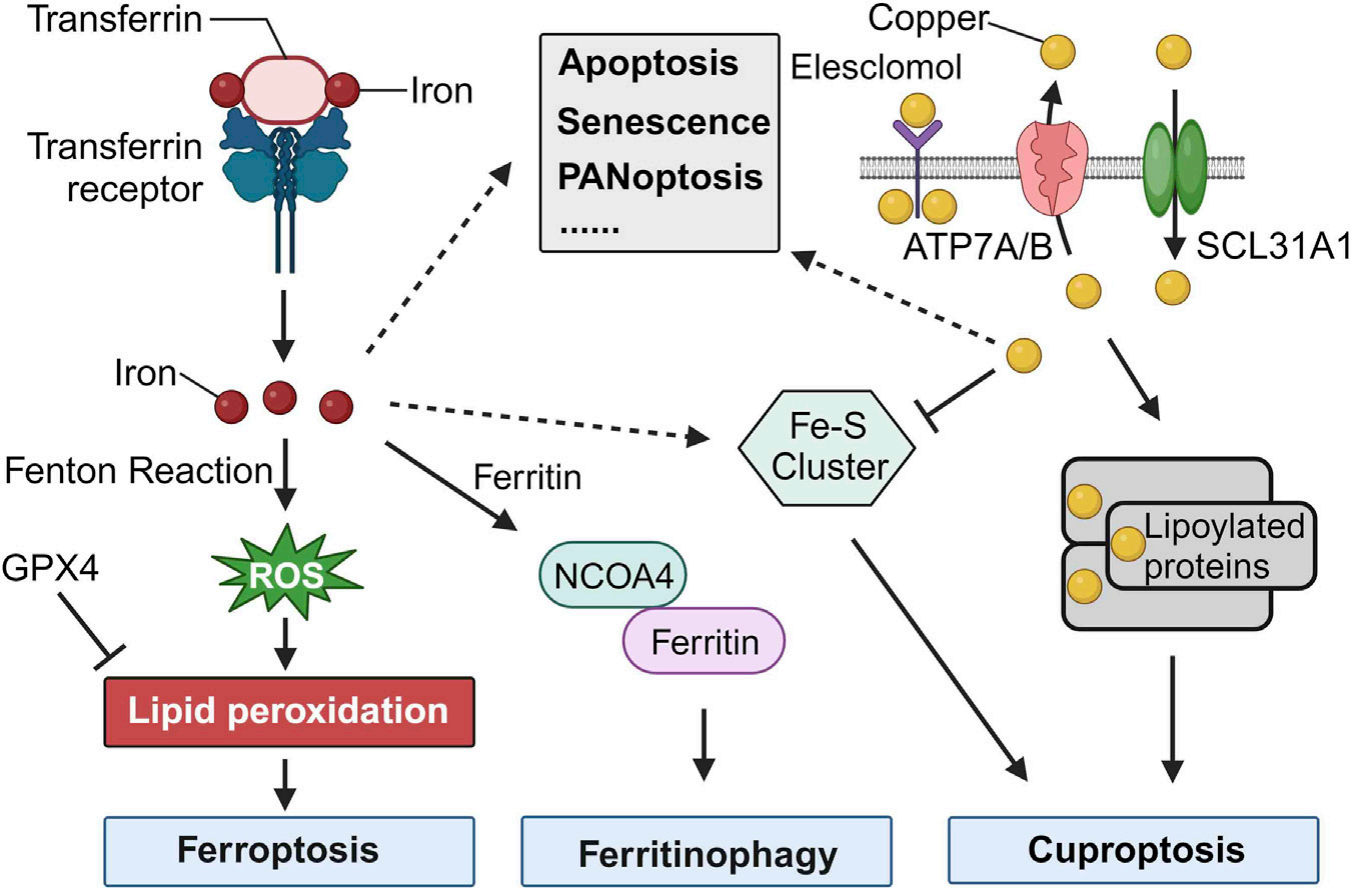
Cell Death Induced by Iron and Copper. The release of iron into the cytosol can lead to the production of reactive oxygen species (ROS) through the Fenton reaction, a chemical reaction that produces hydroxyl radicals from the reaction of hydrogen peroxide and ferrous iron. The accumulation of ROS can lead to lipid peroxidation, which can then trigger ferroptosis. GPX4, an antioxidant defense enzyme, repairs oxidative damage to lipids and is a leading inhibitor of ferroptosis. The increase of iron in the cytosol is regulated by increased import (via the transferrin receptor), decreased export (via ferroportin), and increased iron metabolism. Iron can also be stored in ferritin and delivered to the lysosome for iron release mediated by NCOA4. Increased ferritinophagy contributes to ferroptosis. Additionally, Fe-S cluster formation is a major factor in the initiation of cuproptosis. Copper binding to lipoylated proteins lead to the oligomerization of lipoylated proteins, which induces proteotoxic stress and ultimately results in cuproptosis. Iron and copper also play critical roles in various other types of cell death, such as apoptosis, senescence, and PANoptosis.

Iron, a critical component in many cellular functions, plays an indispensable role in DNA synthesis and other intracellular processes (Puig et al., 2017). However, its accumulation can lead to a unique type of cell death known as ferroptosis, which is distinctly iron-dependent and driven by the generation of reactive oxygen species (ROS). This form of cell death is marked by the build-up of lipid peroxidation products and lethal ROS stemming specifically from iron. Key mechanisms in ferroptosis include lipid peroxidation, the activity of the glutathione peroxidase 4 enzyme, and, centrally, iron. Although iron is vital for numerous cellular activities, an overabundance at the cellular level can prove toxic, producing ROS, which in turn can result in ferroptosis, emphasizing the delicate balance required for iron homeostasis (Figure 1).

Traditional Chinese medicine (TCM) has been playing an increasingly important role in the clinical treatment of human diseases. In recent years, there has been a growing body of research on the mechanism of ferroptosis in TCM (Gao et al., 2022). Liu et al. summarized the findings of this research and reviewed the mechanism of TCM-induced ferroptosis. This article mainly introduces the application of ferroptosis in studies of the mechanism of TCM to help clinicians understand the status of TCM therapy for the treatment of ferroptosis-related diseases.

Cancer cells are more susceptible to ferroptosis because they have an increased demand for iron (Hassannia et al., 2019). Ferroptosis is a form of regulated cell death that is triggered and driven by iron-mediated oxidative mechanisms (Lei et al., 2022). Meng et al. review the relationship between ferroptosis, driven by lipid peroxidation due to iron overload, and its impact on tumor metastasis, emphasizing the pivotal role of circRNAs and their interaction with miRNAs, offering insights for future targeted cancer therapies. Conversely, Yang et al. concentrate on the influence of ferroptosis and associated non-coding RNAs in the context of hepatocellular carcinoma, detailing their function in tumorigenesis, progression, and drug resistance, suggesting avenues for novel treatment strategies and prognostic markers.

Ferroptosis and its related regulators could potentially be used as indicators of cancer. Cheng et al. constructed an ferroptosis-related eight-gene prognostic model for Lung Adenocarcinoma, demonstrating that patients with high-risk scores experienced poorer survival outcomes, diminished immune cell infiltration, and potential resistance to immune checkpoint inhibitors, but displayed heightened sensitivity to therapeutic agents such as Afatinib, Erlotinib, Gefitinib, and Osimertinib. On the other hand, Hong et al. uncovered a distinct Lung Adenocarcinoma subtype based on ferroptosis-associated lncRNAs, with LINC01572 pinpointed as a potential suppressor of ferroptosis in Lung Adenocarcinoma. Huang et al. discovered that the upregulation of the iron-modulating gene, HAMP, linked to promoter hypomethylation and immune checkpoint factors, effectively differentiated clear cell renal cell carcinoma from normal renal tissue. This upregulation served as a significant predictor of unfavorable survival outcomes, indicating its promise as an immunotherapeutic biomarker for cancer patients. On the other hand, the ferroptosis could be a promising new target for the treatment. Abu-Serie demonstrated that a nanocomplex made of ferrous oxide nanoparticles combined with diethyldithiocarbamate displayed superior ability to target and eliminate metastatic liver cancer cells due to enhanced ferroptotic activity, with potential as a promising metastatic liver cancer treatment. Zheng et al. found that polyphyllin I inhibit gastric cancer growth by inducing ferroptosis, with its effects potentially mediated by the NRF2/FTH1 pathway, indicating a potential therapeutic strategy for gastric cancer.

## Ion-induced cuproptosis in cancer

Cuproptosis is a novel form of programmed cell death that is triggered by copper dependence and is distinct from other forms of cell death. It involves the accumulation of fatty acylated proteins, reduction of iron-sulfur clusters, and modulation of mitochondrial functions. Copper plays a significant role in regulating cellular processes, and cuproptosis has been linked to various diseases, including cancers (Xie et al., 2023). While cuproptosis is primarily driven by copper, there seems to be evidence that Fe-S clusters play a role in the mechanism, indicating an intersection between iron and cuproptosis (Figure 1) (Tang et al., 2022). Kong and Sun presented a review highlighting the role of copper accumulation in cancer cells and its significance in tumor growth and metastasis, while also examining the therapeutic potential of targeting copper metabolism for enhanced cancer treatment outcomes.

Long non-coding RNAs (lncRNAs) are a class of RNA molecules that, while not coding for proteins, have been increasingly recognized for their pivotal roles in regulating gene expression, cellular processes, and pathways (Zhang et al., 2023). Aberrant expression of lncRNAs has been implicated in cancer initiation, progression, and metastasis, marking them as potential diagnostic markers and therapeutic targets (Peng et al., 2023). Xie et al. identified cuproptosis-related lncRNA signatures in kidney renal papillary cell carcinoma, emphasizing their ability to predict prognosis and discern immune responses based on unique copper concentrations; concurrently, Chen et al. employed similar lncRNA signatures, specifically spotlighting LEF1-AS1, to craft a prognostic model for glioma progression and therapeutic potential; meanwhile, Li et al. applied these insights to colon adenocarcinoma, developing a model using five distinct lncRNAs to adeptly forecast survival and inform treatment strategies. Branching out from lncRNAs, Yang et al. studied the DLD gene in cuproptosis across cancers, finding its expression influenced prognosis and was linked with immune activity and mitochondrial processes, highlighting its potential as a therapeutic target and prognosis marker.

Delving into the intricate relationships between cuproptosis-related genes and cancer prognosis, several studies have recently shed light on novel predictive models and therapeutic insights. Liu et al. developed an effective predictive model for colon adenocarcinoma using seven specific Epigenetic Associated Cuproptosis genes that effectively categorized patients into risk groups. This model revealed significant differences in survival rates, tumor mutations, and responsiveness to chemotherapy, underscoring the potential of these genes in refining treatment strategies and deepening the understanding of cuproptosis. Sun et al. developed a novel copper metabolism-based risk model for stomach adenocarcinoma, which predicts patient survival and immunotherapy response, underscoring the potential of these genes as prognostic markers and therapeutic guides. Zhu et al. identified three copper-related subtypes in lung adenocarcinoma, with the genes SLC31A1 and ATP7B playing key roles in T-cell exhaustion and prognosis; their findings also introduced a score that aids in predicting treatment responses. Zhao et al. analyzed cuproptosis patterns in hepatocellular carcinoma. They identified two distinct cuproptosis-related mutational patterns that influence the presence of immune cells in the tumor and patient survival. High-risk scores were associated with poorer outcomes and reduced treatment response. Specific genes were found to promote cancer cell growth and resistance to copper-induced death. The study highlights the importance of cuproptosis in hepatocellular carcinoma progression and its potential to guide treatment decisions.

## PANoptosis and other ion-related cell death

Iron, fundamental to numerous biological processes, is integral to proteins and enzymes facilitating oxygen transport, DNA synthesis, and myriad cellular functions. One of its paramount roles is forming iron-sulfur (Fe-S) clusters, intricate assemblies of iron and sulfur atoms present in a wide range of proteins across various organisms. These Fe-S clusters are instrumental in directing various programmed cell death pathways, notably in ferroptosis and cuproptosis (Figure 1). Their essentiality in apoptosis and PANoptosis also highlights their influence over the mitochondrial microenvironment, further underscoring their impact on cellular death processes (Zhang et al., 2022).

PANoptosis refers to a type of cell death characterized by the interplay between pyroptosis, apoptosis, and necroptosis. This process has been identified as being deeply involved in cancer and immunity (Zhu et al., 2023). Wei et al. identified PANoptosis-related gene FADD as a significant risk factor in lung cancer, with high levels affecting treatment responses, and found that inhibiting FADD reduced lung cancer cell proliferation, suggesting its potential as a therapeutic target in lung cancer management. Liu et al. identified a five-gene risk score (PLCG1, PYCARD, CASP8, NOD1, and NOD2) from Pyroptosis-related genes, which effectively predicts targeted therapy and immunotherapy responses in soft tissue sarcoma patients.

Ion homeostasis plays a vital role in cell health, and any imbalance can lead to various cell death forms, impacting cancer development and therapeutic approaches. Ferroptosis, an iron-centric process, has shown significant promise in treating specific cancers like lung adenocarcinoma. In contrast, cuproptosis, copper-dependent cell death, has been associated with new predictive models and therapeutic strategies. The extensive role of iron, especially its involvement in iron-sulfur clusters, is showcased in its influence on several cell death pathways, including the intricate PANoptosis. As research progresses in these areas, new therapeutic directions emerge, offering more effective strategies against cancer.
